# AlloSigMA 2: paving the way to designing allosteric effectors and to exploring allosteric effects of mutations

**DOI:** 10.1093/nar/gkaa338

**Published:** 2020-05-11

**Authors:** Zhen Wah Tan, Enrico Guarnera, Wei-Ven Tee, Igor N Berezovsky

**Affiliations:** Bioinformatics Institute, Agency for Science, Technology and Research (A*STAR), 30 Biopolis Street, #07-01, Matrix, 138671, Singapore; Bioinformatics Institute, Agency for Science, Technology and Research (A*STAR), 30 Biopolis Street, #07-01, Matrix, 138671, Singapore; Bioinformatics Institute, Agency for Science, Technology and Research (A*STAR), 30 Biopolis Street, #07-01, Matrix, 138671, Singapore; Department of Biological Sciences (DBS), National University of Singapore (NUS), 8 Medical Drive, 117579, Singapore; Bioinformatics Institute, Agency for Science, Technology and Research (A*STAR), 30 Biopolis Street, #07-01, Matrix, 138671, Singapore; Department of Biological Sciences (DBS), National University of Singapore (NUS), 8 Medical Drive, 117579, Singapore

## Abstract

The AlloSigMA 2 server provides an interactive platform for exploring the allosteric signaling caused by ligand binding and/or mutations, for analyzing the allosteric effects of mutations and for detecting potential cancer drivers and pathogenic nsSNPs. It can also be used for searching latent allosteric sites and for computationally designing allosteric effectors for these sites with required agonist/antagonist activity. The server is based on the implementation of the Structure-Based Statistical Mechanical Model of Allostery (SBSMMA), which allows one to evaluate the allosteric free energy as a result of the perturbation at per-residue resolution. The Allosteric Signaling Map (ASM) providing a comprehensive residue-by-residue allosteric control over the protein activity can be obtained for any structure of interest. The Allosteric Probing Map (APM), in turn, allows one to perform the fragment-based-like computational design experiment aimed at finding leads for potential allosteric effectors. The server can be instrumental in elucidating of allosteric mechanisms and actions of allosteric mutations, and in the efforts on design of new elements of allosteric control. The server is freely available at: http://allosigma.bii.a-star.edu.sg

## INTRODUCTION

While the concept of allosteric drugs is a relatively new paradigm in drug design, it already shows important achievements and even greater promises supported by a number of approved medicines and numerous drug candidates in development and clinical trials (1–4). High specificity and selectivity of allosteric drugs allow them to be instrumental in solving challenges of the emerging precision medicine (5–7). The important role of allosteric mechanisms in originating latent drivers expanding the cancer mutational landscape (8,9) and in the action of non-synonymous single-nucleotide polymorphisms (nsSNPs, (10)), in general, call for their careful consideration (11,12) in diagnostics and gene therapy applications (3,7).

There is a growing understanding that allosteric drugs and their binding sites have physicochemical characteristics and modes of binding distinct from those of orthosteric medicines (1,2). This prompts researchers to develop completely new libraries of allosteric compounds, to modify algorithms for searching the allosteric sites and for finding efficient allosteric site-effector pairs, and to develop new models for formalizing the mechanisms of the allosteric signaling (3). To this end, the power of modern high-throughput experimental techniques can still be complemented by the computational approaches, which are, in turn, being boosted by the recent advances in the artificial intelligence (AI) techniques (13). Successful utilization of the AI learning power requires, however, presence of basic molecular models in which complexity of biomolecules and their functions would be encoded via libraries of simple elements and interactions between them, allowing the multistep deep and reinforcement learning.

There are several theoretical models (14–16) and corresponding web-applications (2,17–19), allowing to predict allosteric sites (18,20,21), communication between the allosteric and functional sites (15,17,19), ligand–protein interactions (22), and providing tools for the design of allosteric modulators (23). Though the spectrum of approaches used in these models spans from the statistical considerations of sequences and structures of proteins and ligands to simplified physical models, they all possess limited design capabilities (2,14,17–19,21–23). Our goal here is to provide a computational framework for the advance in design of allosteric effectors for natural and newly found allosteric sites. To this end, we implemented our Structure-Based Statistical Mechanical Model of Allostery (SBSMMA), which can provide a comprehensive allosteric control over the protein activity at per-residue resolution (20,24,25). While in the original AlloSigMA (26) we aimed at allowing users to analyze the causality and to estimate the energetics of allosteric effects originated by ligand binding and/or mutations, AlloSigMA 2 is a step forward towards design of site-effector pairs that would provide required allosteric regulation. The server produces Allosteric Signaling Maps (ASMs) and Allosteric Probing Maps (APMs) of proteins that contain exhaustive data on the allosteric signaling from every protein residue to all other residues of the protein (ASM) and on the effect of probing the structure with a small probe that binds to three-residue segments of the protein chain (APM), respectively. The ASMs and APMs can be used as an input in the investigation of the effects of individual mutations and their combinations, in the search for the candidate allosteric sites, and for building the candidate effectors that provide required allosteric modulation of protein activity.

## THEORETICAL BACKGROUND, BENCHMARKING AND IMPLEMENTATION

### Structure-based statistical mechanical model of allostery

We implemented here our Structure-Based Statistical Mechanical Model of Allostery (SBSMMA, (18,20,24)), which allows the calculation of allosteric free energy, or work exerted on the regulated sites and residues as a result of a perturbation caused by ligand binding and/or mutations.

The energy function of a protein with perturbations represented by $C\alpha$ harmonic model for approximating the global dynamics of the protein near equilibrium reads

(1) $$\begin{equation*}\begin{array}{@{}*{1}{l}@{}} {{E^{(P)}}({\boldsymbol{r}} - {{\boldsymbol{r}}^0},S,m) = {\sum _{\left\langle {i,j} \right\rangle ,\;i \ne m}}{k_{ij}}{{\left( {{d_{ij}} - d_{ij}^0} \right)}^2} + \alpha {\sum _{\left\langle {i,j} \right\rangle \in S}}k{{\left( {{d_{ij}} - d_{ij}^0} \right)}^2}}\\ {\quad \quad \quad \quad \quad \quad \quad \quad \quad + \theta {\sum _{\left\langle {m,j} \right\rangle }}{k_{mj}}{{\left( {{d_{mj}} - d_{mj}^0} \right)}^2}} \end{array}\end{equation*}$$

where the first term depicts unbound/wild-type conformational state of the protein, second and third—describe effects of ligand binding and mutations, respectively (for details, see (24)). We consider two types of mutations in SBSMMA: stabilizing (UP, ↑; mimics a residue substitution with bulkier amino acids) and destabilizing (DOWN, ↓; substitutions to small Ala/Gly-like residues).

A microscopic allosteric potential evaluates the elastic work that is exerted on a particular residue *i* as a function of the change of displacement of its neighbors, which is caused by the normal modes associated with the unperturbed $( 0 )$ and perturbed $( P )$ protein configurational states

(2) $$\begin{equation*}{U_i}(\sigma ) = \frac{1}{2}\mathop \sum \limits_\mu {\varepsilon _{\mu ,i}}\sigma _\mu ^2,\quad \quad {\varepsilon _{\mu ,i}} = \mathop \sum \limits_j {\left| {{{\boldsymbol{e}}_{\mu ,i}} - {{\boldsymbol{e}}_{\mu ,j}}} \right|^2}\end{equation*}$$

where $\sigma \; = \;( {{\sigma _1}, \ldots ,{\sigma _\mu }, \ldots } )$ is the amplitude of the change of displacement (${r_i}( \sigma ) - \;r_i^0 = \mathop \sum \limits_\mu {\sigma _\mu }{e_\mu }\;$) and parameters $\varepsilon _{\mu ,i}$ are defined from the sets of orthonormal eigenvectors $e_\mu ^{( 0 )}$ and $e_\mu ^{( P )}$ obtained by diagonalizing the Hessian matrices of the original and perturbed protein energy functions (Equation 1), respectively.

The per-residue partition function is obtained by integrating over the configurational ensemble given by all possible displacements $\sigma$ of a residue *i*, allowing to calculate the per-residue partition function and free energy

(3) $$\begin{equation*}\begin{array}{@{}*{1}{l}@{}} {{z_i} = \smallint d\sigma {e^{ - {U_i}(\sigma )/{k_B}T}} = \mathop \prod \limits_\mu {{\left( {\pi \frac{{2{k_B}T}}{{{\varepsilon _{\mu ,i}}}}} \right)}^{{\raise0.7ex\hbox{$1$} \!\mathord{\left/ {\vphantom {1 2}}\right.} \!\lower0.7ex\hbox{$2$}}}},}\\ {{g_i} = \frac{1}{2}{k_B}T\mathop \sum \limits_\mu {\rm{ln\ }}{\varepsilon _{\mu ,i}} + \ const} \end{array}\end{equation*}$$

Comparison of two protein states, unbound/wild-type reference state (0) and perturbed (*P*) states provides the per-residue free energy difference of the allosteric effects as a result of the perturbation by ligand(s) binding and/or mutation(s)

(4) $$\begin{equation*}\Delta {\rm{\;}}g_i^{\left( P \right)} = \frac{1}{2}{\rm{\;}}{k_B}T\mathop \sum \limits_\mu \ln \frac{{\varepsilon _{\mu ,i}^{\left( P \right)}}}{{\varepsilon _{\mu ,i}^{\left( 0 \right)}}}{\rm{\;}}\end{equation*}$$

which evaluates the change in configurational work acting on a residue *i* because of the perturbation. The background free allosteric effect, allosteric modulation evaluates the free energy difference from its mean value over the protein chain

(5) $$\begin{equation*}{\rm{\Delta }}h_i^{\left( P \right)} = {\rm{\Delta }}g_i^{\left( P \right)} - {\left\langle {{\rm{\Delta }}g_i^{\left( P \right)}} \right\rangle _{Chain}}\;\end{equation*}$$

Allosteric modulation close to zero indicates that the response at the residue/site of interest is similar to the protein-average $\Delta g_i^{( P )}$ value, i.e. to the background effect on the whole protein.

A positive value $\Delta h_i^{( P )} >0$ indicates that work exerted on residue *i* may induce conformational changes caused by the perturbation. A negative $\Delta h_i^{( P )} < 0$ value shows a stabilization of residue *i*, preventing it from the conformational change. The effect of a perturbation on the functional sites of interest are obtained as an average over all the residues belonging to the site.

We define the *allosteric modulation range*$\Delta h_i^{( {m \downarrow \uparrow } )}$, which is a generic descriptor of the strength of allosteric signal on residue $i$ originated from substitutions of the residue $m$ that can be calculated for any residue position $i$ of the protein

(6) $$\begin{equation*}\Delta \;h_i^{\left( {m \downarrow \uparrow } \right)} = \;\Delta h_i^{\left( {m \uparrow } \right)} - \Delta h_i^{\left( {m \downarrow } \right)}\;\end{equation*}$$

The allosteric modulation range evaluates the maximal potential value of the allosteric signal that can be caused by the mutation from the smallest (Ala/Gly-like) to the bulkiest (for example, Phe or Trp) residues. The calculation of the allosteric modulation range is used for deriving the Allosteric Signaling Map (ASM) of the protein, which is the exhaustive description of the allosteric response in the protein upon residue-by-residue perturbation.

We also consider Allosteric Probing Map (APM), in which the allosteric modulation on residue $i$ is originated by the binding of the small probe (described by the second term of the energy function in Equation 1) to a three-residue segment of the protein modelled sequentially from residue 1 to residue N-2 for a protein chain of N residues. The allosteric modulation caused by the probe is also evaluated as a background free effect:

(7) $$\begin{equation*}{\rm{\Delta }}h_i^{\left( {Probe} \right)} = {\rm{\Delta }}g_i^{\left( {Probe} \right)} - {\left\langle {{\rm{\Delta }}g_i^{\left( {Probe} \right)}} \right\rangle _{Chain}}\end{equation*}$$

Noteworthy, the SBSMMA (20) implemented in the server has several important advantages as well as some drawbacks. The lack of atomic details in the $C\alpha$ harmonic model obviously affects the quality of the allosteric effects' estimation. Indirect modeling of the binding effect and simplified consideration of two-type mutations are also limitations as structure of the ligand (or mutated residue) or the actual set of interactions in the binding sites and upon mutations are not explicitly considered. On the other hand, the indirect way of mimicking the ligand binding is a generic framework that can be applied to different proteins without any preliminary knowledge of the allosteric sites. The crude way of considering only two types of mutations allows us to perform and exhaustive scanning of complete proteins and to use a generic measure, allosteric modulation range (Equation 6), for estimating allosteric signalling caused by the potential mutation of any protein residues regardless of the amino acid type in the native structure. The model could be also improved by introducing the sequence dependence of the energy function, which is currently work in progress. Concluding this discussion on the balance between the model's limitations and advantages, in the trade-off between approximation used in the model and its computational cost the SBSMMA provides great advantages of: (i) allowing the high-throughput analysis of many structures and (ii) exhaustive accounting of allosteric signalling at per-residue resolution (ASMs and APMs). In case of necessity for in-depth analysis with atomic resolution the harmonic model should be replaced with the MD simulations followed by the principal component analysis of the covariance matrix that would provide an input (principal components instead of normal modes) for the statistical-mechanical consideration. This change will come, however, at the price of high computational cost, which prevents the massive and quick analysis.

### Benchmarking of the predictive power of the structure-based statistical mechanical model of allostery (SBSMMA)

**Figure 1.**
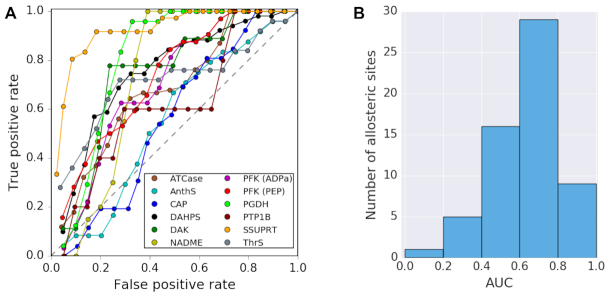
Benchmarking of the prediction of allosteric sites using the reverse perturbation in the SBSMMA. (**A**) Receiver operating characteristic (ROC) curves for 11 proteins from the classical set of allosteric proteins; (**B**) area under curves (AUC) for the extended set of 52 allosteric proteins (11 proteins from the classical set and 41 from the ASBench database (27)) with 60 known allosteric sites.

We have previously shown that SBSMMA model can be used for predictions of potential allosteric sites by simulating ligand binding to protein functional sites, *i.e*. by reversing the allosteric communication (25). We hypothesized that binding of a substrate and/or a cofactor at functional sites would lead to a large increase of the configurational work exerted on allosteric sites thereby allowing their identification. As there can be any number of unknown latent allosteric sites, the quality of the allosteric site prediction can be only estimated on the basis of the predictions of known allosteric sites. Therefore, we collected a set of 11 well-studied allosteric proteins with experimentally validated allosteric and functional sites, dubbed as the classical set (Supplementary File, Table S1), to benchmark the predictive power of the approach. Functional and allosteric sites are delineated by residues located within 4.5 Å from respective ligands in crystal structures. We use the operational definition of allosteric sites (proximity <2%), which requires that functional and allosteric sites do not overlap (25). Only allosteric sites satisfying above definition were used in the benchmark experiment. We simulated ligand binding to functional sites of proteins from the classical set using AlloSigMA 2 server. From the distributions of per-residue allosteric modulation obtained upon the perturbation, receiver operating characteristic (ROC) curves are plotted by calculating the true and false positive rates for a series of bins, starting from the top 5% of the positive range of the distribution and followed by 10%, 15% and so on with a 5% step. A residue is considered as the true positive if it belongs to a known allosteric site, whereas a false positive indicates otherwise. The ROC curves show that for most allosteric sites, the true positive rate increases more rapidly than the false positive rate, indicating that most of the residues of known allosteric sites exhibit a large increase of free energy as an allosteric response upon perturbing the functional sites (Figure 1A). We further complemented the classical set with an additional set of 41 proteins with 48 allosteric sites (Supplementary File, Table S2) collected from the ASBench database (27). Similarly, we measured the ROC curves for the additional set and calculated the area under the ROC curves (AUC). Figure 1B shows that large AUC values are obtained for both protein sets consist of a total of 52 proteins with 60 allosteric sites, indicating the high predictive power for the allosteric sites.

### Implementation

The AlloSigMA 2 server is powered by the Python Flask library (http://palletsprojects.com/p/flask/), with 3D structure visualizations rendered using the highly efficient JavaScript PV library (http://dx.doi.org/10.5281/zenodo.20980), per-residue free energy changes and ASMs/APMs are rendered using the JavaScript Plotly library (http://plot.ly), and the data-driven visualization of the effects of individual mutations or small probe bindings is powered by the D3.js library (http://d3js.org). The C_α_ harmonic model implemented in the Molecular Modeling Toolkit (MMTK, (28)) is used for the normal mode analysis. Ten lowest frequency normal modes are considered in calculations (20). The server is interfaced with the Protein Databank (29) and PDBePISA (30). The system can process structures with up to 1800 amino acid residues, and up to 26 protein chains. Processing in the first mode can be done on-line, while results in the ASM and APM options will be produced separately with e-mail notification to user upon the job completion. To improve user experience, we have also implemented a job queueing system using the Python Celery library (http://www.celeryproject.org), allowing users to be notified on the job progress via email (if provided). Interactive visualization of ASMs and APMs is possible for structures up to 1800 residues, as it is limited by the capacity of web-browsers. For larger structures, users will be provided with a PDF image of the computed ASM and APM along with the downloadable package containing results of the analysis and corresponding matrices, which can be further analyzed according to the users’ needs.

## DESCRIPTION OF THE SERVER

### Input and preprocessing

**Figure 2.**
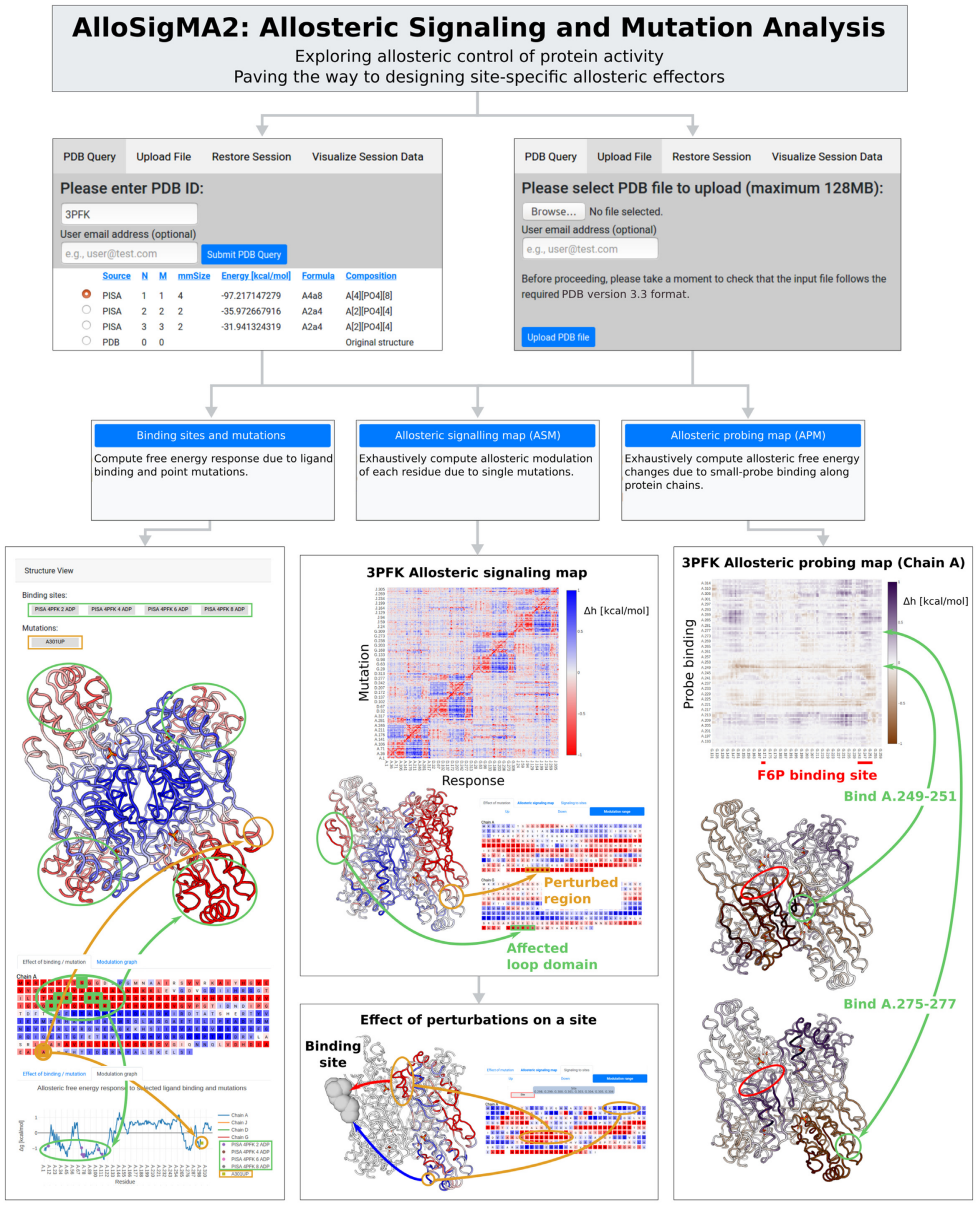
Flowchart of the navigation through the AlloSigMA 2 server. The structure can be fetched from the PDB or uploaded by the user directly. There are three modes of the operation in the server: ’Binding Sites and Mutations‘, ’Allosteric Signaling Maps‘ and ’Allosteric Probing Maps‘. Example of the outputs of these modes are illustrated in corresponding columns.

For the server input the user can provide either the PDB ID of an existing protein X-ray structure or upload an individual file with protein coordinates in the standard PDB format (Figure 2, two blocks in the top row). The preprocessing starts from the ordered list of biological assemblies in the PISA database, according to the solvation free energy gain upon assembly formation (30). If no assembly is found, the structure may be fetched from the Protein Databank as is. Ten best matching homologs (99% sequence identity) generated in the VAST server (31) are used for compiling a comprehensive list of binding sites, which are then mapped to the correct chains of the considered protein structure. In the original work on SBSMMA (20), we showed a qualitative similarity in the allosteric communication between allosteric sites and functional sites of different apo and holo protein structures. However, for practical purposes, we recommend users to use apo form (if available), as it is natural to observe effects on functional sites as a result of ligand binding in the apo form.

### Output of the server

The second row in the flowchart representing AlloSigMA 2 functionality shows three modes of the analysis available in the server: ‘Binding sites and mutations’, ‘Allosteric Signaling Map (ASM)’ and ‘Allosteric Probing Map (APM)’.

The first mode, ‘Binding sites and mutations’, allows users to evaluate and visualize dynamical changes on the protein upon perturbations in form of ligand(s) binding and/or stabilizing(UP)/destabilizing(DOWN) mutation(s). This part of the server is an update of the original AlloSigMA, color-coding the allosteric signaling dynamical changes on both 3D structure and sequence representations: conformational changes are prevented in regions marked by the gradient of red (negative allosteric free energy change) and originated in regions marked by gradient of blue (work is performed on these residues – positive allosteric free energy). To start processing the user should select binding sites of interest and/or to make the choice of the UP-mutations (stabilizing) and DOWN-mutations (destabilizing). It is possible to consider only some of the protein chains in case if it is only required to analyze a part of the protein complex/oligomer (see the on-line ‘Tutorial’ for details). In this server update, we have improved the dashboard interface, using the JavaScript PV library (http://dx.doi.org/10.5281/zenodo.20980), to enable smooth visualization of free energy changes, the binding pockets and mutated residues on the 3D protein structure. Per-residue free energy changes along protein chains are plotted interactively using the Plotly library (http://plot.ly) for greater ease of zooming in to analyze specific regions of interest. In the left column of Figure 2, we illustrate the effects of having 4 ADP ligands bound on PFK (green) and of UP mutation at the residue A.301 (yellow). The color-coded representation of the per-residue allosteric effect on the sequence shows parts of the structure with corresponding changes of the dynamics. The bottom chart contains a graph representation of the allosteric signaling as a result of the perturbation (here, four bound ligands marked by green and one mutation - orange). Noteworthy, analyzing outputs the user should remember that while values exceeding ${k_B}T$ should be regarded as a strong manifestation of the allosteric communication, combinations of the low-values allosteric responses may result in significant allosteric modulation in homogeneously affected protein regions.

The second mode of operation, ‘Allosteric Signaling Map (ASM)’, is an instrument that allows to obtain a comprehensive control over the protein activity. The ASM is a matrix that contains an exhaustive information on the allosteric communication in the protein, where every row shows the allosteric signal from the residues on y-axis to all other residues of the protein (x-axis) represented by the allosteric modulation range ($\Delta h_i^{( {m \downarrow \uparrow } )}$) – the maximal potential value of the allosteric effect as a result of the substitution from the smallest (Ala/Gly like) to the bulkiest amino acid (e.g. Trp or Phe). Using the ASM, it is possible to monitor allosteric signaling at per-residue resolution, quantifying, thus, effects of individual mutations and of substitutions in regulatory exosites on the allosteric signaling. In the context of what types of mutations are more prone to induce certain allosteric response in the protein, researcher can first consult with ASM for UP and DOWN mutations, which would guide the user to decide on required type of mutation. The ASM can also be used for evaluating potential allosteric signaling from the sites designated by the user. An example of ASM built for the phosphofructokinase (3PFK) is shown in the central column (Allosteric Signaling Map) of Figure 2, which highlights major patterns of negative and positive modulations that indicates the domain and oligomeric structures of the protein. For example, in tetrameric PFK each monomer consists of two domains strongly interacting with each other via last quarter of the second domain (3PFK ASM, Figure 2). In the ‘Effect of mutation’ tab, users may select one or more mutations to visualize the allosteric effects of these mutations on the structure and sequence. The structure-sequence pair below the ASM show how it can be used for the analysis of the effect of perturbations of several residues (yellow region in chain A) can allosterically affect dynamics in a loop domain of another chain (G.298–306, green). Further, to find mutations that can have a significant modulating effect on specific domains or binding sites, the ‘Signaling to sites’ tab was included to enable users to see the total free energy change on selected domains when individual residues are mutated. The bottom structure-sequence pair in the central column illustrates opposite effects of mutations on the loop domain depending on the locations of mutations on the chain A of PFK. In this case, both structure and sequence are colored according to what allosteric signal (positive modulation – blue; negative – red) is observed on the investigated binding site.

The third mode of operation, ‘Allosteric probing map (APM)’, computes the effect of small-probe binding along the protein chain, simulating the ligand binding to three consecutive residues along the protein chains. We simulate the probe binding using three consecutive residues, which is the minimal required size to describe the composition of binding sites that provide sufficient strength to identify the communication to functional sites originating from all possible allosteric sites. At the same time, three-residue probes should give a readout on the effect of binding small fragments/moieties of lead molecules without sacrificing the resolution, compared to probing larger ones. On the basis of the exhaustive scanning of three-residues from the Allosteric Probing Maps, the user can then extend/merge the probes or define a binding site based on the three-residues probe in order to explore the allosteric effect. By mimicking binding of small ligand, the APMs allow users to scan through the protein for latent allosteric sites that may regulate protein activity, facilitating the fragment-based design of new allosteric effectors that can provide high specificity. Using the ‘Effect of binding’ tab, the user can select probe sites and visualize the effect of the perturbation across the protein: conformational changes in some regions indicated by purple or prevention of them marked by dark orange. The matrix in the right column ‘Allosteric Probing Map (APM)’ of Figure 2 is a zoom-in of the APM, showing an example how small-probe binding events (green) in chain A can differently affect the stability of the F6P binding site on chain G (red). Modelled binding of a small probe to two sampled sites A.249–251 and A.275–277 produce opposite effects on the F6P site (Supplementary File, Figure S1): while the former tends to stabilize the local structure and restrict access to the binding site, the latter initiates conformational changes in the pocket, potentially allowing for easier access and binding of the substrate.

### Example of the virtual experiment on the fragment-based design of allosteric effector using ASM and APM

In order to explain how the ASM–APM combination can be used for computational design of allosteric effectors, we describe here a virtual experiment on building two small ligands originating opposite allosteric signaling to the catalytic site. We use here a classical allosteric protein, phosphofructokinase (PFK), which performs the key step of glycolysis via phosphorylation of fructose-6-phosphate (F6P) into fructose-1,6-bisphosphate. The substrate F6P binds to a cleft between the two domains of a subunit, and the 6-phosphate group interacts with His249 and Arg252 from one subunit, and Arg162 and Arg243 from the neighboring subunit. We investigated the allosteric signaling to these four residues, developing a simple protocol for the fragment-based design of the allosteric effector. Our goal here is to: first, start from the analysis of the ASM and to find locations in the protein, from which strong allosteric signal can propagate to residues of the F6P site upon perturbation; second, to explore these locations with a small probe that mimics a binding to three consecutive residues in the protein chain and to find those that cause the strongest allosteric modulation on the F6P site; third, to complement the initial probes with additional elements that further modify the allosteric signal. We seek for mutations/probes where none of the residues involved are located within 11 Å from each of the four responding residues (B.162, B.243, D.249 and D.252) – the cutoff for being not in contact and act allosterically used in the SBSMMA (20,24,25).

**Figure 3.**
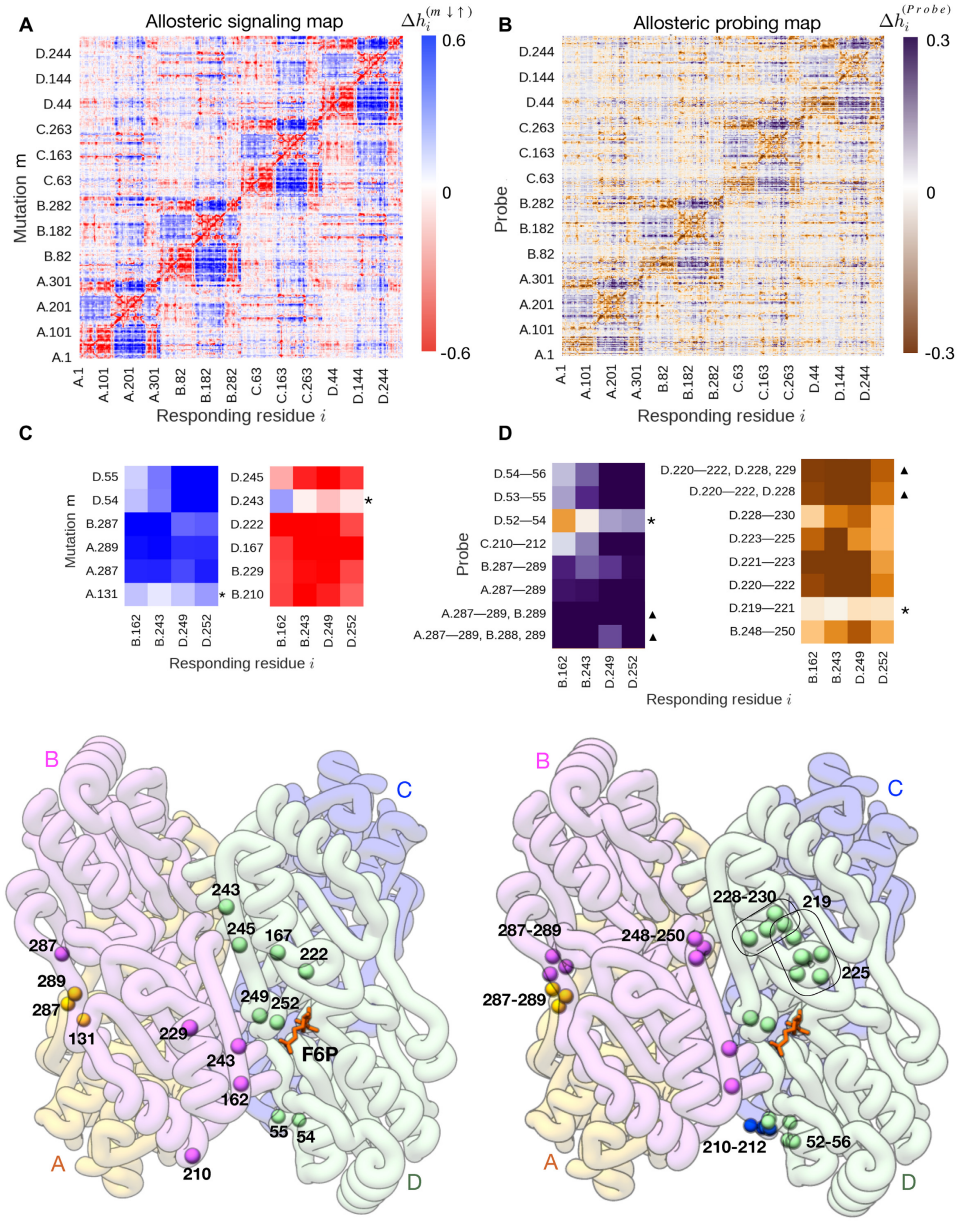
Example of the virtual computational experiment on the design of the lead molecule for allosteric effector using combination of ASM and APM. (**A**) ASM of the phosphofructokinase (PFK); (**B**) APM of the PFK; (**C**) strongest allosteric modulation on the residues of the F6P binding site as a result of mutations (illustrated on the structure below); (**D**) strongest allosteric modulation on the residues of the F6P binding site as a result of the probe binding, complemented by the effect of the modified probe binding (illustrated by framed residues on the structure below).

Figure 3 illustrates realization of the above protocol. On the first step, we ranked allosteric modulation ranges in the ASM of protein (Figure 3A, PDB_ID: 3pfk) and found five residues that originate the strongest positive and negative allosteric modulation on the four residues of the F6P binding site (B.162, B.243, D.249 and D.252). Negative controls located in the vicinity of selected ones, but showing much weaker response are marked by asterisk (Figure 3C). The second step, screening with a small probe produced the APM (Figure 3b), which reveals fragments with the strongest effects, such as A.287–289 and B.287–289 that originate positive and 220–222, 221–223 and 223–225 that cause negative allosteric modulation on the four residues of the F6P binding site (Figure 3D). We also checked the probes bound to the ‘control region’ (D.52–54 and D.219–221) and found that they indeed did not originate any significant allosteric signaling (Figure 3d). On the final step, we simulated the fragment-based design by considering the contributions from additional elements added in the probe. We found that addition of one (B.289) or two (B.288,289) adjacent residues to the probed binding site (A.287–289) strengthens the positive allosteric modulation on the F6P site (Figure 3D, marked by triangle). At the same time, addition of residue D.228 or pair D.228, 229 to the probed site D.220–222 strengthens the negative modulation on the F6P site (Figure 3D, marked by triangle). These observations show the tunability of allosteric signals that can be achieved via the ‘fragment-based’ effector design. Obtaining the shapes of probes that interact with corresponding probe-binding sites on the protein can provide the initial leads of the effector molecules to be further modified.

## CONCLUSIONS

The AlloSigMA 2 server is an update of the original AlloSigMA server (26), which is a response to the growing demand on the computational modelling and screening of allosteric drugs. As we discussed it recently elsewhere (3), the quest for allosteric drugs is as strong as challenging because of the distinctive characteristics of the allosteric sites, effectors and their modes of interactions and actions (2,3,18). We, therefore, proposed to use recently developed very basic physical model, structure-based statistical mechanical model of allostery (SBSMMA, (20,24,25)), which allows to take protein activity under comprehensive allosteric control at per-residue resolution (24). Complementing the old options that allow to evaluate the energetics of allosteric signaling caused by ligand binding and/or mutations (26), we provide here two new modes of operation – Allosteric Signaling Map (ASM) and Allosteric Probing Map (APM). The ASM of a protein contains an exhaustive residue-by-residue description of the allosteric signaling, where the allosteric effect of substitution on every protein position is calculated for all other residues of the protein. In addition to the demonstrated above way of using the ASM data for prediction of the allosteric tuning of the protein activity (5,6), prediction of the latent cancer drivers (8–10) and of the effects of nsSNPs is another important area of ASM application (7,10). The APM, in turn, describes the complete picture of allosteric modulation as a result of the binding of small probe that interacts with three-residue segments of the protein. We show how starting from the big picture of allosteric signaling in the protein presented in ASM, one can find potential allosteric sites that would provide required mode and strength of the allosteric communication. Then, turning to APM, the user can evaluate an effect of the ligand binding as a result of the screening with a small probe designated to interact with a three-residue segment of the protein chain followed by the adjustment of the required effect with added (on the basis of ASM data) interactions. Resulting pattern on the protein structure will serve as a template for sketching a lead candidate of potential allosteric drug. We would like to emphasize that the exhaustive and, at the same time, very basic nature of ASMs and APMs allow to obtain the high-throughput data on many proteins of different types and, then, to use it as an input to the deep and reinforcement learning approaches (13). We believe, therefore, that AlloSigMA 2 can be of great help for: (i) computational evaluation and planning of experimental efforts on the allosteric control of protein activity and on design of allosteric effectors; (ii) for obtaining the ASM and APM data, which will be indispensable in future efforts on the prediction of allosteric effects of nsSNPs and cancer drivers, and, of course, for the advances in the AI-based design of allosteric drugs.

## Supplementary Material

gkaa338_Supplemental_File

## Notes

Present address: Enrico Guarnera, Global Analytical Pharmaceutical Science and Innovation, Merck KGaA, Via Luigi Einaudi, 11, Guidonia Montecelio, 00012 Rome, Italy.
