# Supplementary material for: AlloSigMA 2: paving the way to designing allosteric effectors and to exploring allosteric effects of mutations
Source: Nucleic Acids Res. 2020 May 11;48(W1):W116–24. doi: 10.1093/nar/gkaa338 (PMC7319554; doi:10.1093/nar/gkaa338)

| **Protein** | **PDB** | **Functional site** | **Allosteric site** | **AUC** |
| --- | --- | --- | --- | --- |
| Anthranilate synthase | 1i7q | GLU | TRP | 0.55 |
| Aspartate carbamoyltransferase | 1d09 | PAL | ATP-CTP | 0.66 |
| Catabolite activator protein | 1o3q | DNA | cAMP | 0.55 |
| 3-deoxy-D-arabino-heptulosonate-7-phosphate synthase | 1kfl | PEP | PHE | 0.73 |
| Dimeric arginine kinase | 3ju5 | ATP/ARG | ARG | 0.73 |
| NAD-dependent malic enzyme | 1efk | NAD | FUM | 0.73 |
| Phosphofructokinase | 3pfk | F6P/ADPf | ADPa/PEP | 0.69/0.71 |
| D-3-phosphoglycerate dehydrogenase | 1yba | AKG/NAD | SER | 0.79 |
| Protein tyrosine phosphatase 1B | 2hnp | BPM | 892 | 0.62 |
| Uracil phosphoribosyltransferase | 1xtu | UMP | CTP | 0.87 |
| Threonine synthase | 1e5x | PLP | SAM | 0.69 |

Supplementary Table 1

The classical set of 11 proteins used for benchmarking.

| **Protein** | **PDB** | **Functional site** | **Allosteric site** | **AUC** |
| --- | --- | --- | --- | --- |
| Acetyltransferase Pat | 4avb | ACO | CMP | 0.67 |
| Amino-acid acetyltransferase | 3d2p | COA | ARG | 0.41 |
| Anaerobic ribonucleoside-triphosphate reductase | 1h78 | DCP | DCP | 0.76 |
| Androgen receptor (Homo sapiens) | 2pio | DHT | AV6 | 0.70 |
| Androgen receptor (Mus musculus) | 2qpy | DHT | 4HY | 0.71 |
| Antithrombin III (Homo sapiens) | 1t1f | RCL | NTP | 0.72 |
| ATP phosphoribosyltransferase | 1nh8 | AMP | HIS | 0.69 |
| cAMP receptor protein | 3d0s | DNA | CMP | 0.56 |
| Chorismate mutase | 3csm | TSA | TRP | 0.16 |
| Farnesyl pyrophosphate synthase | 2qis | RIS | 3N1 | 0.47 |
| Fatty acid metabolism regulator protein | 1hw1 | DNA | COA | 0.72 |
| Fructose-1,6- bisphosphatase 1 (Shigella boydii) | 2q8m | FBP | AMP | 0.79 |
| Fructose-1,6- bisphosphatase 1 (Sus scrofa) | 1fbp | FBP | PFE | 0.63 |
| Glucose-1-phosphate thymidylyltransferase 1 | 1mc3 | TTP | TMP | 0.63 |
| Glutamate dehydrogenase 1, mitochondrial | 1nr7 | GLU | GWD | 0.59 |
| Glycogen phosphorylase, liver form | 2ati | GLC | AVE/CP4 | 0.79/0.89 |
| Glycogen phosphorylase, muscle form (Homo sapiens) | 1z8d | GLC | AMP | 0.69 |
| Glycogen phosphorylase, muscle form (Oryctolagus cuniculus) | 2skc | GLC | FRY/QUE | 0.88/0.24 |
| HTH-type transcriptional repressor PurR | 1qp0 | DNA | HPA | 0.69 |
| Isocitrate dehydrogenase [NADP], mitochondrial | 4ja8 | NDP | 1K9 | 0.5 |
| Isocitrate dehydrogenase kinase/phosphatase | 3eps | ATP | AMP | 0.51 |
| L-lactate dehydrogenase | 1ldn | NAD | FBP | 0.79 |
| L-lactate dehydrogenase 2 | 1lld | NAD | FBP | 0.78 |
| Lactose operon repressor | 1efa | DNA | NPF | 0.64 |
| Leukotriene A-4 hydrolase | 5fwq | BES | 692 | 0.87 |
| Lysine-sensitive aspartokinase 3 | 2j0w | ADP | LYS | 0.53 |
| Mitogen-activated protein kinase 14 | 1wfc | L9G | 0O8 | 0.8 |
| Mitogen-activatedprotein kinase 8 | 1ukh | 537 | 46A | 0.36 |
| Multifunctional 2- oxoglutarate metabolism enzyme | 2y0p | TD7 | ACO | 0.61 |
| Myosin-2 heavy chain | 1yv3 | ADP | PBQ | 0.58 |
| NAD(P)-dependent glyceraldehyde-3- phosphate dehydrogenase | 1uxu | NAP | AMP | 0.84 |
| Ornithine decarboxylase | 1njj | ORX | GET | 0.8 |
| Parathion hydrolase | 1qw7 | CO | EBP | 0.33 |
| Plasminogen activator inhibitor 1 | 1oc0 | Bchain | 96P | 0.47 |
| Pyruvate dehydrogenase kinase isozyme 2 | 2bu2 | ATP | TF2/TF3/TF4 | 0.85/0.22/0.34 |
| Pyruvate kinase PKLR | 2vgb | PGA | FBP | 0.75 |
| Pyruvate kinase PKM | 3gqy | TLA | FBP/NZT/SER | 0.59/0.83/0.58 |
| Ribonucleoside- diphosphate reductase 1 subunit alpha | 4r1r | GDP | ATP/TTP | 0.79/0.69 |
| Tyrosine-protein kinase ABL1 (Homo sapiens) | 3pyy | STI | 3YY | 0.58 |
| Tyrosine-protein kinase ABL1 (Mus musculus) | 3k5v | STI | STJ | 0.54 |
| Uridylate kinase | 4a7x | UDP | GTP | 0.54 |

Supplementary Table 2

The additional benchmark set of 41 proteins

Figure S1. Effects on the F6P site originated by the single mutation and probe binding to the segment with this single mutation


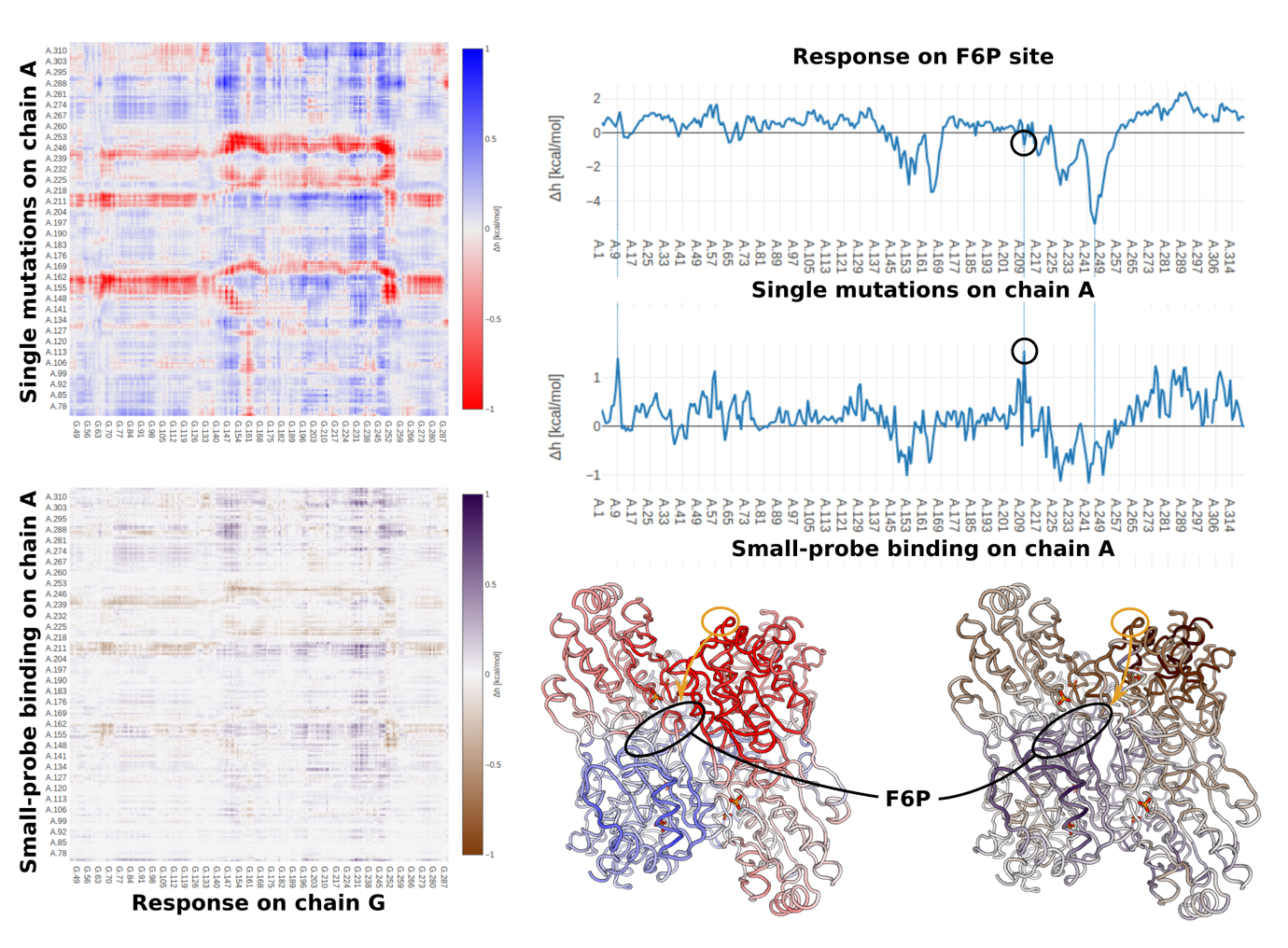

Supplement: gkaa338_Supplemental_File [file gkaa338_supplemental_file.docx]
